# Cancer and Pregnancy: A Comprehensive Review

**DOI:** 10.3390/cancers13123048

**Published:** 2021-06-18

**Authors:** Roxana Schwab, Katharina Anic, Annette Hasenburg

**Affiliations:** Department of Obstetrics and Women’s Health, University Medical Center of the Johannes Gutenberg University Mainz, 55131 Mainz, Germany; katharina.anic@unimedizin-mainz.de (K.A.); annette.hasenburg@unimedizin-mainz.de (A.H.)

**Keywords:** cancer, pregnancy, diagnostic procedures, therapeutic procedures, fetal outcome

## Abstract

**Simple Summary:**

The co-occurrence of cancer and pregnancy is fortunately a rare event. It is a challenging situation for all involved parties, as the risks and benefits of oncological treatments should be balanced for both the mother and the offspring. This work offers guidance to clinicians regarding the choice of diagnostic and therapeutic procedures, as well as regarding the management of the pregnancy.

**Abstract:**

Cancer diagnosis and treatment in pregnant women is a challenging situation. A multidisciplinary network of specialists is required to guide both, the expecting mother and the unborn child through the diagnostic workup and the cytotoxic therapy, by balancing the respective risks and benefits. Tumor entity, stage, biology and gestational week at diagnosis determine the appropriate approach. As premature delivery emerged as one of the main risk factors for adverse long-term outcome of the progeny, it should be avoided, if reasonable from the oncological perspective. This article offers a comprehensive review with respect to the various aspects of cancer in pregnancy.

## 1. Introduction

Cancer associated with pregnancy or delivery is a rare diagnosis with an incidence of 1/1000–2000 pregnancies [1,2]. Nevertheless, the prevalence of cancer and pregnancy is increasing due to changed sociodemographic variables, such as increased maternal age due to postponing the start of building a family [3]. Moreover, new screening methods for fetal aneuploidies, such as noninvasive prenatal testing (NIPT), accidentally may help to detect occult malignancies in pregnant women by revealing aberrant genome representation profiles such as somatic copy number variations [4].

Breast cancer is the most common malignancy during pregnancy with an incidence of 1/3000 [5], followed by other entities, such as cervical cancer, hematological malignancies, ovarian cancer, colorectal cancer and melanoma [1,2]. When a malignancy is suspected, the diagnostic workup should be initiated immediately. After diagnosis, the pregnant women should be managed within a multidisciplinary team. Medical decisions and counselling of pregnant women and their partners should follow ethical guidelines. A close and efficient cooperation with distinct medical specialties is required, beginning with the correct diagnosis and close monitoring of the mother and the fetus up to an extended follow-up period. This is essential for providing optimal care for the expecting parents and the unborn child, by carefully balancing the risks and benefits of both sides.

When cancer is diagnosed during pregnancy, a very challenging situation emerges for the expectant mother, the unborn child and the medical team. Evidence-based information regarding diagnostic and treatment plans, as well as regarding the maternal outcome, is still limited, compared to the vast data available for non-pregnant cancer patients [6]. Nevertheless, new insights and evidence-based information regarding the management of cancer in pregnancy, as well as several international consensus meetings of leading experts, have paved the way for a more standardized approach to assure the best outcome for mother and child [3,6,7]. As a result, the management of pregnant women has improved over time. Nowadays, oncological therapy is favored over termination of pregnancy. Consequently, a 20-year international cohort study of 1170 patients showed that more live births were counted over time [3,8]. Nevertheless, preterm delivery, a well-recognized major risk factor for adverse neonatal outcomes in pregnancies complicated by cancer, should be avoided [3,8].

This comprehensive review aims to give an overview over the current standard procedures regarding diagnostic, therapeutic modalities and challenges of pregnant cancer patients, as well as to discuss the challenges regarding the unborn child.

## 2. Therapeutic Principles in Women with Cancer in Pregnancy

In general, treatment of pregnant patients with cancer should follow the guidelines and recommendations of those for non-pregnant patients [6]. Nevertheless, the week of gestation at diagnosis, the expected prognosis of the mother, the wishes and concerns of the expectant parents, as well as ethical considerations, should be carefully taken into account for each patient.

### 2.1. Diagnostic Workup during Pregnancy

In most cancer patients, adequate imaging procedures are crucial elements in the work-up algorithm of tumor stage evaluation and verification of therapy effectiveness. The diagnostic work-up should follow the guidelines and recommendations for non-pregnant patients with cancer [6].

If reasonable, diagnostic procedures without ionizing radiation should be preferred. When available, whole-body (diffusion-weighted) MRI should be advised. It allows radiation-free imaging of the entire body during one examination and ensures sensitive cancer detection during pregnancy [6,9]. The use of gadolinium contrast material should be avoided, as it passes the placenta, although, to date, no negative effects were observed in humans [10].

Ionizing radiation, as used for radiographs or CT scans, may lead to stochastic effects, such as cellular damage on DNA level causing germ cell mutations. The fetal dose of ionizing radiation can be estimated either by the medical physicist or by placing a thermoluminescent dosimeter to the surface of the patient, the fetal dose being approximately one-third of the entrance dose to the mother [10]. There is no threshold value to stochastic effects and the specific risks may vary with gestational ages and with differing radiation exposure. Nevertheless, the margins of safety for in utero exposure to ionizing radiation were set to 50 mGy, but fetal risks seem to be low up to a dose of 100 mGy [10,11]. The excess risk of childhood cancer after intrauterine exposure to 10 mGy is 1:1667 and after exposure to 50 mGy 1:334 [12]. The mental functioning seemed not to be affected at doses below 100 mGy even during organogenesis [13]. The fetal radiation dose is dependent on the diagnostic procedure and the involved organs or body parts. The fetal radiation dose is extremely low in chest x-rays procedures and extremity radiographs (<0.01 mGy) [14], as well as in CT scans of the chest (0.02 mGy), while direct abdominal or pelvic CT scans deliver a much higher ionizing radiation dose (10 to 50 mGy) [10,14].

Higher doses of ionizing radiation lead to deterministic, non-stochastic effects, with the threshold level set to 150 mGy and termination of pregnancy should be considered at a cumulative radiation dose exceeding this level [10]. Gross congenital malformations were increased at doses exceeding 200 mGy [11], and the lifetime risk of cancer development in individuals exposed in utero to levels above this threshold was approximately 3% [10].

In sum, diagnostic procedures with ionizing radiation can be performed after balancing the maternal and fetal risks and benefits. Unnecessary procedures should be avoided and irradiation dose reducing techniques should be implemented [2,10]. Moreover, intravenous iodinated contrast material seems to be safe when applied to pregnant women, but the thyroid function of the progeny should be assessed after birth [10].

### 2.2. Surgery during Pregnancy

Depending on the tumor entity, localization, stage and prognosis, surgery during pregnancy may be inevitable. Surgery can be performed on all non-abdominal or non-genital organs with safety if special precautions are used.

As up to 0.5–2% of pregnant women will undergo non-obstetric surgery, there is growing evidence, that even abdominal surgery during pregnancy can be performed with relative safety [6]. This is crucial since surgery is an important and integral part of the treatment of gynecological malignancies. Nevertheless, surgery of gynecological organs during pregnancy should be performed by an experienced team [15]. Laparoscopy during the first and second trimester of the pregnancy is feasible, if the maximum procedure time will not exceed 90 min, the intra-abdominal pressure will not exceed 10–13 mmHg, if the first trocar is introduced by open incision, and if a highly experienced minimal-invasive surgeon will perform the procedure [15], otherwise mid-line laparotomy should be considered. Despite all precautions, negative obstetric outcomes, such as preterm delivery, miscarriage and fetal distress may result following surgery during pregnancy [6]. Perioperatively, the hemodynamic parameters of the mother should be monitored thoroughly, to assure optimal fetal supply [6]. Moreover, the mother has an increased risk for aspiration, due to the pregnancy-associated gastroesophageal reflux [6].

Surgery in breast cancer patients, the predominant tumor entity diagnosed during pregnancy, is a challenge. While breast-conserving surgery requires adjuvant radiotherapy, and as radiotherapy should be avoided during pregnancy, neoadjuvant chemotherapy is an option for these patients to delay surgery until delivery (Table 1) [16,17]. If surgery cannot be delayed until delivery, mastectomy is an option, depending on the week of gestation and the intended time of prolonging the pregnancy. Moreover, as chemotherapy should be avoided during the first trimester, mastectomy may be the only option for treatment of patients diagnosed during this vulnerable period of organogenesis (Table 1) [18]. Tissue expander insertion seem to be a feasible procedure during pregnancy [17]. As with other above mentioned surgical procedures, the 2nd trimester is recommended for surgical procedures (Table 1). Axillary lymph node staging using lymphoscintigraphy with 99 m-Tc sulfur colloid seems to be safe all throughout the pregnancy and should be preferred over axillary lymph node dissection (Table 1) [16,17].

In case of ovarian masses, and when a malignancy is not highly suspected, intraabdominal surgery should be postponed until the second trimester, when the documented abortion rate was 8.7% after adnexal surgery [19]. Another multicenter retrospective analysis assessing patients with malignant ovarian masses during pregnancy described a live birth rate after surgery of 78.82% [20]. Fortunately and due to routine antenatal ultrasound, adnexal malignancies are usually detected at an early stage during pregnancy [21]. In cases with borderline tumors of the ovary, conservative surgery should be performed during pregnancy and additional staging surgery should be performed after delivery (Table 1) [7,22]. In epithelial ovarian cancer and if the disease is limited to the ovary, surgical staging can be performed either before 22 weeks of gestation or after delivery (Table 1) [7]. Hysterectomy has no proven benefit with respect to survival. Moreover, hysterectomy did not improve outcome in premenopausal patients with stage II epithelial ovarian cancer [23,24]. Chemotherapy should be performed during pregnancy, if indicated (Table 1) [7]. In advanced stages of ovarian cancer with peritoneal spread, either termination of pregnancy or neoadjuvant chemotherapy should be considered before 22 weeks of gestation (Table 1) [7,20,25,26]. After 22 weeks of gestation, chemotherapy should be offered with consecutive debulking surgery after delivery (Table 1) [7,20,25,26].

Non-epithelial ovarian tumors are very rare during pregnancy. Surgical staging procedures with pregnancy preservation can be performed during the first and second trimester (Table 1) [27]. In malignant ovarian germ cell tumors during pregnancy, Zong et al. suggested to postpone chemotherapy until delivery [27]. In pregnant women, chemotherapy for non-epithelial ovarian tumors should be preferably performed following the EP regimen (etoposide and platinum) or the standard chemotherapy for epithelial ovarian cancer (carboplatin and paclitaxel) rather than with BEP (bleomycin, etoposide and platinum) (Table 1) [26,28].

Treatment of cervical cancer during pregnancy is very challenging. Pregnancy-preserving management is a feasible option in tumors stage IB1 or less, while laparoscopic lymph node staging should be offered until 22 weeks of gestation and beginning with tumor stage IA1 with lymphovascular space invasion or in higher tumor stages (Table 1) [7]. Surgical options in low stage cervical cancer during pregnancy are either conization or simple trachelectomy, depending on tumor stage (Table 1) [29], as conization after negative lymph node evaluation seems to be a safe and feasible conservative option in early-stage cervical cancer patients [30,31]. A high abortion rate of 32% was described in these patients following conization or simple trachelectomy [15]. Radical trachelectomy should be avoided, if reasonable. Nevertheless, even in women treated with radical trachelectomy, 80% live birth rates after vaginal radical were reported [25]. After 22 weeks of gestation, either delayed surgical oncological treatment until delivery or the application of neoadjuvant chemotherapy (preferably cisplatin to avoid side effects) should be discussed with the patients (Table 1) [7,32]. In stages IB2 and less than 22 weeks of gestation, either pelvic lymphadenectomy with chemotherapy or follow-up, or neoadjuvant chemotherapy should be offered (Table 1) [7]. The application of neoadjuvant chemotherapy is the only pregnancy-preserving option in stage IB3 [7]. Cesarian section should be recommended in patients with cervical cancer during pregnancy, to prevent tumor spread during cervical ripening and delivery (Table 1) [29]. Moreover, Arakawa et al. reported two cases of vertical tumor transmission during vaginal delivery from mothers with cervical cancer to their children [33]. High tumor stages (IB3 or higher) or lymph node invasion should be an indication for pregnancy termination (Table 1) [7,15]. In pregnancy non-preserving management, feticide can be performed before initiation of surgical treatment or before chemoradiotherapy (Table 1) [7].

Vulvar cancer is extremely rare during pregnancy. In these patients, local excision with lymph node staging by locally injected technetium-99 using a short treatment protocol is feasible during pregnancy (Table 1) [7,25]. Radiotherapy of vulvar or vaginal cancer should not be performed during pregnancy. In cases with metastatic lymph node disease and need for radiotherapy, therapy delay for 6 to 8 weeks can be offered after balancing the risks and benefits (Table 1) [7]. In cases with high risk for groin recurrence, immediate radiotherapy of the inguinal region should be performed and thus, depending on the week of gestation, either delivery or termination of the pregnancy should be discussed (Table 1) [7]. Cesarian section is the preferred mode of delivery after surgery of the vulvar to avoid wound dehiscence (Table 1) [7].

Only a few cases of vaginal cancer during pregnancy are described to date [7]. Local excision should be performed, but advanced stages would require disease management with radiotherapy (Table 1) [7]. In these cases, either delivery or termination of the pregnancy should be considered depending on the week of gestation and the wishes of the patients (Table 1) [7].

### 2.3. Chemotherapy during Pregnancy

Chemotherapy is an integral part of oncological therapy of various cancer entities, and thus an integral part of oncological therapy during pregnancy. Nevertheless, specific cautions with respect to the gestational week, with consequences to fetal development and risks, as mentioned in the paragraph below, should be observed.

Due to physiological changes during pregnancy, drug absorption, distribution, metabolism and drug excretion may differ from non-pregnant women, resulting in a dilutionary effect of the cytotoxic drugs by altered bioavailability, and increased hepatic and renal elimination [6,34]. Fortunately, these effects seemed not to impede the effects of the chemotherapy with respect to DSF and OS in a population of breast cancer patients [35].

Chemotherapy should be avoided during the first trimester but is considered relatively safe to use during the 2nd and 3rd trimester [2]. Cardonick et al. proposed a delay of chemotherapy until 16.6 weeks of gestation, if reasonable from oncological perspective [36]. The antimetabolites cytarabine and fluorouracil can be applied during the 2nd and 3rd trimester, but methotrexate should be avoided during the entire pregnancy, due to multiple birth defects [2]. The alkylating agents cyclophosphamide and dacarbazine were evaluated to be safe [2], while busulfan showed negative effects in the development of the central nervous system in animal models [37]. The anthracyclines doxorubicin and epirubicin are considered to be safe during the 2nd and 3rd trimester, nevertheless, idarubicin and daunorubicin should be avoided [2]. The vinca alkaloids vinblastine and vincristine, as well as in limited cases vinorelbine, are considered safe in the 2nd and 3rd trimester, and vinblastine in the first trimester, as well [2]. The taxanes paclitaxel and docetaxel were reported to be safe in the 2nd and 3rd trimester [2]. The platinum agents carboplatin and cisplatin are considered to be safe in the 2nd and 3rd trimester, while limited data are available for oxaliplatin [2].

The use of supportive medication is indispensable during treatment with cytotoxic drugs. The use of antiemetics is well studied, as they are also prescribed in the common condition of hyperemesis gravidarum. The effect of ondansetron was superior to the treatment with metoclopramide and the combination of doxylamine and pyridoxine and the use during pregnancy seemed not to be associated with an increased rate of malformations [38,39,40]. In contrast, the use of glucocorticoids during the first trimester was associated with an increased risk of oral cavity abnormalities [41]. To reverse the myelosuppressive effect of certain cytotoxic drugs, growth factors, such as Granulocyte Colony-Stimulating Factor (GCSF), can be applied during pregnancy and safety data are reassuring [42]. Anemia is usually treated with a combination of iron, folate and Vitamin B-12, but severe anemia may require transfusion of red blood cells.

Information regarding drug safety, when applied during pregnancy and lactation, can be extracted from the drug information sheet. For the United States, the Federal Drug Administration (FDA) used to assign a specific category to each drug (A, B, C, D and X) according to the known adverse effects to the fetus and the neonate. In 2015, the FDA implemented the Pregnancy Lactation Labeling Rule and removed the previous categories in favor of updated information for helping clinicians to discuss in detail the potential risks and benefits of each administered drug with the expectant mother [43]. For clinicians practicing in Australia, the “Prescribing medicines in pregnancy database” administered by the Department of Health of the Australian Government is another source of information [44]. This database labels the drugs in distinct categories (A, B1, B2, B3, C, D, X) [44], depending on the observed risk to the developing embryo and fetus. Another database, called “Embryotox”, is available for German-speaking countries [45].

Possible effects of cytotoxic therapy on the development of the fetus are described in the paragraph below (Section 3).

### 2.4. Biologicals/Targeted Therapies during Pregnancy

During the last 25 years, cancer treatment developed into a personalized approach. Targeted therapies, specifically addressing the molecular and immunological drivers of tumorigenesis revolutionized oncological treatment. Nevertheless, data regarding treatment of challenging patient groups, such as pregnant women, are still limited, as only a few prospective randomized studies are available [46,47]. The understanding of the impact of targeted therapies on implantation, embryogenesis and on fetal development is mainly based on single case reports. An overview of the available experience with targeted therapies in pregnant women is displayed in Table 2.

As with every medication during pregnancy, a thorough consideration must be made with respect to the profit-loss ratio regarding the oncological benefits of the mother and the health risks of the offspring. In general, anti-HER2 therapy should not be applied during pregnancy, but no malformations were reported in cases of accidentally administration of trastuzumab during the 1st trimester [48,49]. The use of trastuzumab may be discussed in high-risk situations and may be administered with close fetal monitoring [17]. Rituximab, the most frequently used targeted therapy during pregnancy (Table 2) may be considered as a therapeutic option when indicated to treat a life-threatening maternal malignant or hematological disease [50]. Discontinuation of treatment with targeted therapies should be recommended with an inadequate maternal oncological response or when serious fetal adverse outcomes are registered [46,47]. Nevertheless, as evidence and experience with the clinical use and safety are scarce, it is commonly recommended to postpone the use of targeted therapies until after delivery [7].

**Table 2:** Targeted therapies during pregnancy.

Targeted Therapy	Mode of Action	Placental Passage in Humans	Physiological Role of Target in Human Embryonal and Fetal Development	Exposure in 1st Trimester in Humans	Exposure in 2nd and 3rd Trimester in Humans	Evidence Level
Trastuzumab	Monoclonal IgG1 antibody against human epidermal growth factor (HER2) receptors [47]	-low during 1. Trimester -increasing during second and third trimester [46]	Implantation Cardiac and neural development [46,47]	25% spontaneous abortion - No congenital malformations [47] No mandatory pregnancy interruption when exposed during the first trimester [46]	Oligohydramnios (68.1%) (reversible) Fetal renal failure Fetal death due to multiorgan failure due to prematurity, anhydramnios or oligohydramnios (17.3%) [5,46]	34 cases (Case reports) [5,47]
Other anti-Her-2 agents (lapatinib, pertuzumab and T-DM1)	-	No data	Implantation Cardiac and neural development [47]	Lapatinib: three cases with no congenital malformation [47,49]	No data	Three cases (lapatinib) [49]
Bevacizumab	VEGF-specific mAb	No data	Vasculogenesis and angiogenesis of the placenta and in normal fetal development [47]	No data regarding systemic exposure [47] Intravitreal exposure followed by abortion in some cases [47]	No data regarding systemic exposure [47] Intravitreal injection with no adverse effects [47]	No reports for systemic application in pregnancy [46,47]
Rituximab	mAb IgG targeting the surface antigen CD 20	transplacental passage increases with gestational age, reaching the maximum during the last 4 weeks of gestation. [46]	Hematopoiesis (lymphocytes) [51]	No congenital malformations [50] Miscarriage rate 21% [51]	Cytopenia (63% of neonates at full term) -complete neonate recovery from hematotox within 6 months [50]	A total of 253 pregnancies were reported, but pregnancy outcome was available for 153 pregnancies only [46,50]
Imatinib	TKI targeting the bcr-abl tyrosine kinase [47]	Placental transfer documented [47]	-fetal organogenesis [47]	-Major malformations 11% [47] -spontaneous abortion 12.1% [47]	-no major or minor malformations [47]	Case reports (*n* > 180) [47]
EGFR inhibitors (erlotinib, gefitinib, afatinib and cetuximab)	EGF receptor	Placental transfer documented [47]	Conception Implantation Embryonic development	No congenital malformation [47]	No congenital malformation [47]	Sparse to no data [47]
ATRA (tretinoin)	Carboxylic acid form of vitamin A [47]	Placental transfer documented [47]	Fetal development [47]	Spontaneous abortion [47]	Abnormal cardiac function [47]	Case reports [47]
Interferon-a	cytokine	Insignificant placental transfer [47]	Organogenesis [47,52]	2% major malformations (combination therapy with imatinib) [47]	No data	Case reports [47]
Dasatanib	Second-generation TKIs	Placental transfer [47]	Organogenesis [47]	No congenital malformation (three cases) [47] Hydrops fetalis (one case)	No data	Three cases [47]
Nilotinib	Second-generation TKIs	No data	Organogenesis [47]	One case, no congenital malformation [47]	No data	One case [47]
Vemurafenib	BRAF-inhibitor	Placental transfer documented [47]	Cardio-faciocutaneous development [47]	No data [47]	No major malformations (one case) [47]	
PARP inhibitors (Niraparib, Rucaparib, Olaparib)	Poly adenosine diphosphate [ADP]-ribose polymerase (PARP) inhibitor	No data [53]	No data [53]	No data [53]	No data [53]	No data [53]
Anti-PD-1/PD-L1	Immune checkpoint inhibitors	No data [54]	Maintaining normal fetal tolerance [55]	One case report without spontaneous abortion [56]	No data [54]	One case report [56]

IgG = immune globulin G; FDA = federal drug administration; HER2 = human epidermal growth factor receptor 2; T-DM1 = transtuzumab emtansine; mAB = mouse antibody; VEGF = vascular endothelial growth factor TKIs = tyrosine kinases inhibitors; bcr-abl = fusion between the Abelson tyrosine kinase gene and the break point cluster gene, EGFR = epidermal growth factor receptor; ATRA = alltrans retinoic acid; BRAF = gene encoding the protein B-Raf; PARP = Poly adenosine diphosphate [ADP]-ribose polymerase; PD-1 = programmed death protein 1; PD-L1 = programmed death-ligand 1 protein.2.5.

### 2.5. Hormonal Treatment during Pregnancy

To date, hormonal therapy is contraindicated during pregnancy, as these drugs either block estrogen production or interfere with estrogen pathways and may negatively impact the developing embryonic, fetal and placental tissues [2]. With respect to tamoxifen, only case reports are available, showing various birth defects in up to 25% of reported cases after application during and after the first trimester, such as Goldenhar’s syndrome, Pierre-Robin sequence and ambiguous genitalia [2,57]. Aromatase inhibitors seem to exert prenatal developmental toxicity in both the embryonal and the fetal period at threshold exposure levels that were below the human therapeutic dose [58]. New data even described the aromatase inhibitor letrozole as an alternative treatment option to methotrexate in the conservative treatment of ectopic pregnancies, due to its ability to inhibit the estrogen synthetase [59]. To our knowledge, no reports of CDK4/6 application in pregnancy were published to date.

### 2.6. Radiotherapy during Pregnancy

Radiotherapy during pregnancy should be carefully evaluated, as it will deliver higher radiation doses to the progeny than the above-mentioned diagnostic procedures [13]. Radiation doses above 100 mGy and up to 500 mGy during the first trimester led to mental retardation in up to 6% of cases and exposure during the 2nd trimester in 2% of cases, respectively [13]. The risk for mental retardation was lower during the 3rd trimester [13]. Thus, the fetal dose should not exceed 500 mGy as a result of radiotherapy during pregnancy [13]. Special abdominal lead shields are available for reducing the fetal dose during pregnancy by up to 25–50% [60,61]. As the radiation dose is inversely proportional to the distance, radiation therapy of the upper body, e.g., in breast cancer or in Non-Hodgkin or Hodgkin lymphoma should be preferably applied during the first and second trimester, as the distance between the growing fetus and the radiation field narrows with increasing gestational weeks [60,61,62]. Kourinou et al. described, that the estimated fetal dose did not exceed 100 mGy during the first trimester for the assessed radiotherapies in breast, lung and nasopharyngeal cancer [62]. In contrast, the radiation dose to the fetus from treatment for Hodgkin’s lymphoma and lung cancer during the second and third trimesters of gestation, as well as the radiation dose delivered to the fetus during treatment of breast cancer during the third trimester, was found to be higher than 100 mGy [62].

There are a few case reports regarding radiotherapy due to breast cancer during pregnancy. The fetal dose did not exceed 180 mGy during radiotherapy with up to 50 Gy radiation dose of the breast or the chest wall during the first or 2nd trimester, and no intrauterine fetal death was reported [13,63]. The application of an intraoperative boost to the tumor bed may additionally reduce the fetal dose [13]. Candela-Juan et al. published a case report of successful shielded brachytherapy in a patient with breast cancer during the first trimester of pregnancy, and the estimated fetal dose did not reach 50 mGy [61].

Especially radiotherapy of the abdominal or pelvic organs during pregnancy should be avoided [6]. Women treated with radiotherapy due to cervical cancer during the first and second trimester suffered an abortion within 6 weeks after radiation, while 2 women treated with radiotherapy during the 3rd trimester showed an uncomplicated pregnancy course [13].

### 2.7. Prognosis of Women Diagnosed with Cancer in Pregnancy

In oncological patients, the long-term survival of patients is the main goal of treatment. Consequently, one of the major concerns of oncologists is that the pregnancy might negatively influence the prognosis.

To date, reassuring data are available regarding the prognosis of the mother. Stensheim at al. published a population-based cohort study including 42,511 women with a median follow-up of 9 years [64]. For all tumor entities combined, and with adjustment for age, extent of disease and diagnostic period, the hazard ratios for cause-specific death did not significantly differ in pregnant or lactating women compared with the non-pregnant group (HR 1.0) [64]. Similar results were published by Amant et al., as DSF and OS were not significantly different in women with breast cancer diagnosis during pregnancy compared to non-pregnant women (HR 1.3; and HR 1.19, respectively) [35]. Nevertheless, some subgroups, such as patients with HER2+ breast cancer diagnosed in pregnancy seemed to have a poorer prognosis compared to the non-pregnant control-group [65]. Moreover, women diagnosed during lactation or during the first year’s post-partum [66,67] seemed to have a poorer prognosis compared to the non-pregnant control group or those diagnosed during pregnancy. However, pregnant women with malignant melanoma displayed an increased risk of cause-specific death (HR 1.52), while cervical-, thyroid- and ovarian cancer, lymphoma or leukemia did not show a significantly altered cause-specific risk of death (HR 1.23; HR 0.89; HR1.15; HR 4.58; and HR 0.46, respectively) [64].

Maggen et al. published a multicenter, retrospective cohort study with respect to the prognosis of women with Hodgkin lymphoma diagnosed during pregnancy, which showed equal outcomes compared to those of non-pregnant patients [68]. Additionally, early-stage cervical cancer diagnosed in pregnancy seemed also to have a favorable prognosis [69].

Moreover, a retrospective study of 85 pregnant women diagnosed with malignant ovarian tumors during pregnancy, of whom 78.82% were in FIGO stage I, described a global survival rate of 83.53% [20]. Thus, pregnant patients with early-stage malignant ovarian tumors appeared to have favorable outcomes [20].

However, the risk of cause-specific death was significantly increased, if breast or ovarian cancer were diagnosed during lactation (HR 1.59–1.95; HR, 2.23, respectively) [64,67].

Noteworthy, within the population-based cohort study published by Stensheim et al., women previously diagnosed with cancer, who experienced a subsequent pregnancy or a post-cancer pregnancy, showed an approximately 80% lower risk of cause-specific death, enforcing the “healthy mother effect” for women with a post-cancer pregnancy [64].

## 3. Obstetrical Management in Women with Cancer in Pregnancy

### 3.1. Placenta and Pregnancy

The placenta acts as the interface between the maternal and the fetal compartment and thus, the respective blood systems. As such, the placenta is exposed to drugs administered during pregnancy to the mother and, subsequently, its function may be impaired [3,70,71]. Consequently, adverse pregnancy outcomes in women suffering from cancer during pregnancy, such as the high risk of preeclampsia and an elevated risk for fetal growth restriction, may be linked to drug impact on the normal structure and development of the placenta [3]. Studies assessing the direct and indirect impact of cancer drugs on placental physiology are limited. An increased level of oxidative stress in the human placenta after cancer drug exposure with various types of chemotherapeutic agents was recently described by Verheecke et al. [71]. Morphological changes of the macroscopic and microscopic placental architecture, such as villous hypermaturity, distal villous hypoplasia and villous edema were described after exposure to DNA-active cytotoxic drugs during the 2nd and 3rd trimesters of pregnancy [70]. Direct chemotherapy-induced toxic effects caused placental underdevelopment, but other causes, such as malnutrition, stress and immune suppression could not be ruled out [70]. Another in vitro study examining placental tissue explants of 3rd trimester pregnancies, which were exposed to various cytotoxic drugs at therapeutic plasma concentrations (between 1 µM and 100 µM), such as doxorubicin, paclitaxel, cisplatin, carboplatin, crizotinib, gefitinib, imatinib or sunitinib and all cytotoxic drugs but paclitaxel led to reduced cell and tissue viability within 72 h of exposure [72]. In vivo animal studies showed impaired umbilical cord blood flow parameters and reduced placenta efficiency when mice were treated with doxorubicin during gestation [73].

The placenta regulates the level of transfer of drugs to the fetus resulting in different drug concentrations in the fetal compartment, depending on drug molecular structures, and on facilitated and active transport of the specific drugs by the placenta [74]. Animal studies showed decreased concentrations of cytotoxic drugs such as paclitaxel, anthracyclines and vinblastine in the fetal plasma compared to the maternal plasma concentrations [75]. Thus, depending on the administered cytotoxic agent, the placenta acts as a barrier to the fetal compartment. Nevertheless, cytotoxic drugs may lead to major congenital anomalies and mental retardation when administered during the embryonal period and early fetal period, and to functional defects and minor anomalies when administered during the fetal period [76].

In summary, the placenta is on one hand a target organ for cytotoxic drugs, and on the other hand, the placenta enables the transfer of cytotoxic drugs into the fetal compartment, exposing the fetus to these agents.

### 3.2. Fetal Care

Fetal care in pregnancies complicated by a malignant diagnosis of the mother is a challenge for all involved parties, such as the oncologist, the obstetrician specialized in high-risk pregnancies, and the neonatologist [8]. Obstetrical monitoring should follow the respective guidelines for high-risk pregnancies and should consider the possible individual side effects of the administered cytotoxic therapy, the individual fetal development and the specific week of gestation. The goal is to provide optimal care for the expecting parents and the unborn child, by carefully balancing the risks and benefits of the oncological therapy, the fetal development, the optimal timing for delivery and the adequate postnatal care.

#### 3.2.1. First Trimester

Antenatal exposure to cytotoxic therapies may lead to various fetal adverse outcomes, depending on the administered drug and the time of exposure during pregnancy. While exposure during the first trimester was associated with an increased risk for major congenital anomalies (OR 3.9 for birth defects, due to cytotoxic therapy initiated before 16.6 gestational weeks) [36,76], and should therefore be avoided, an increasing data set reinforced the relatively safety of maternal treatment with cytotoxic drugs during the 2nd and 3rd trimester with respect to the malformations of the progeny. The risk of miscarriage was 2% in a large cohort study including more than 1100 women diagnosed with cancer in pregnancy [8] and termination of pregnancy was reported in 9% of pregnancies, primarily due to unfavorable diagnosis of the mother. Women with hematological malignancies during the first trimester should be immediately referred to adequate treatment, which in many cases implicates the termination of pregnancy, due to the embryotoxic side-effects of the therapy [3].

#### 3.2.2. 2nd and 3rd Trimester

Oncological treatment during the 2nd and 3rd trimester may also lead to adverse pregnancy outcomes. Small for gestational age or intrauterine growth restriction infants (SGA or IUGR, defined as birth percentile weight < 10%) were more frequent in this population than in controls (21–28.5% vs. 10%, respectively), with a significantly increased risk (OR 2.99) when chemotherapy was administered before 15 gestational weeks [3,8,36], and after treatment with platinum-based chemotherapy (OR 3.12) [8]. Only 1% of 1170 expectant women with singleton pregnancies suffered a stillbirth during an observation time of 20 years, as reported by an international cohort study [8].

In singleton pregnancies, premature rupture of membranes or premature contractions were frequent, with an incidence of 10% [8]. Nevertheless, the predominant obstetrical complication was premature delivery in 48% of cases, and of those, 88% were iatrogenic and only 12% spontaneous [8]. The risk of preterm birth seemed to decrease in the last decades due to improved obstetrical management (OR 0.91) [8]. Fortunately, the children born with low birth weight seemed to recover the weight difference within a couple of months [77,78]. Due to prematurity, 41% of neonates were admitted to the intensive care unit (ICU), while a strong association of prematurity with maternal gastrointestinal cancer was observed (OR 7.13) [8]. Notably, prematurity but not antenatal chemotherapy administration was associated with lower cognitive developmental scores and with lower IQ scores [77]. Thus, these adverse outcomes of the progeny should be considered during the decisional process for the optimal time point of iatrogenic induced preterm delivery. Additionally, subtle changes in cardiac function were observed in children exposed to antenatal cytotoxic therapy [77].

### 3.3. Delivery

With regard to best fetal outcome, the optimal timing of delivery is crucial. Due to the above-mentioned possible changes in the clinical appearance of the progeny, delivery should be scheduled preferably at term and chemotherapy should be paused after 35 gestational weeks, to allow the recovery of blood parameters of the neonate and the expectant mother in order to avoid perinatal complications [2,79].

In the absence of other obstetric risk factors, vaginal delivery should be favored over cesarian section, as it offers advantages for the mother, such as shorter hospitalization time, less blood loss and decreased risk for infection [6]. Nevertheless, there are special oncological situations, which enforce delivery by cesarian section, for example pregnant women who are suffering from cervical cancer or, in some cases, women presenting with metastasis of the long bones or metastasis of the central nervous system [6]. To exclude metastasis of the placenta, a pathological examination of this organ should be performed after delivery [6].

### 3.4. Lactation after Cancer in Pregnancy

Women diagnosed with cancer during pregnancy may wonder, whether breastfeeding is safe and reasonable. Breastfeeding is a crucial experience with respect to maternal–neonatal bonding [80]. Women experience increased maternal self-confidence by skin-to-skin contact with the newborn and by being able to nurse the child [81]. Additionally, breastfeeding reduced the maternal risk for other diseases in later life, such as diabetes or ovarian cancer. Nevertheless, breastfeeding seems to differentially influence the risk for subsequent breast cancer in specific subgroups of the population. For example, in women of Mexican descent who reported breastfeeding for more than 12 months, the risk of developing triple-negative breast cancer was over twice as high as for developing luminal A tumors [82].

Known benefits for the neonate are the immune-boosting effects of breastfeeding, the establishment of a healthy gut microbiome and a lower risk for developing asthma, type 1 diabetes, food allergies and obesity in later life [83]. Under “normal” circumstances, breastfeeding is recommended as the exclusive source of nutrition for all infants up to the age of 6 months [83]. The number of cycles of chemotherapy, the type of cytotoxic agents, as well as the gestational age at beginning of the cytotoxic therapy seemed to impact the ability to breastfeeding in women who were treated with cytotoxic therapy during pregnancy [80].

Women who have to complete chemotherapy after delivery or for whom the time window to the previous chemotherapy cycle did not reach at least three weeks should be discouraged from breastfeeding, even if the neonate’s toxicity depends on the oral bioavailability of the drug, the neonate’s pharmacokinetics and the amount of milk [84,85]. Transfer of cytotoxic drugs into human milk was reported for several chemotherapeutic agents and their metabolites, such as methotrexate, doxorubicin and cisplatin [85]. The experience with breastfeeding during therapy with targeted agents is based on single case reports. Imatinib and its metabolites were shown to be excreted in human milk [86]. Even though the estimated exposure of the neonate to imatinib was approximately at around 10% of the therapeutic dose and breastfeeding showed no developmental abnormalities after short-term breastfeeding, long-term breastfeeding should be discouraged in these patients [87,88].

Only 55% of 38 women diagnosed with breast cancer during pregnancy and who completed chemotherapy before delivery were able to successfully breastfeed their children [89]. Additionally, 45% to 63.5% of women who received antenatal chemotherapy reported reduced milk production and the need to provide supplemental feeding to their children [80,89,90]. However, women with breast cancer, who may worry that breastfeeding could worsen their health outcome, should be reassured, that breastfeeding is safe and in fact, showed to be protective with respect to cancer recurrence, lowering the risk by 41% compared to age-matched controls [91].

The ability to breastfeed the newborn infant and the satisfaction with breastfeeding seem to influence the mental health of the mother. Reduced milk production was associated with higher levels of psychological distress in women diagnosed with cancer during pregnancy [92]. Additionally, the prevalence of depressive symptoms was lower in women who stated, they were satisfied with breastfeeding.

In summary, breastfeeding has various positive effects on mother and child and especially their relationship. All women diagnosed with cancer during pregnancy and who will not undergo any treatment considered a contraindication to breastfeeding during the postpartum period, should be offered lactation counseling and should be reassured that breastfeeding is safe.

### 3.5. Fetal Follow-Up after Intrauterine Exposure to Cytotoxic Drugs

Additionally, the development of children exposed to antenatal cytotoxic therapy should be assessed by implementing compulsory long-term follow-up procedures [77]. Amant et al., presented reassuring long-term data of children exposed to cytotoxic therapy in utero, that showed that the cognitive outcome was comparable to babies from healthy mothers [77]. The 6-year follow-up of children born to mothers who received oncological treatment during pregnancy revealed, that they showed moderately cognitive developmental alterations [93]. While the full scale IQ was not statistically different from the control group of children who were not exposed to intrauterine cancer treatment, the verbal IQ showed lower values in the study group [93]. Memory span, short-term memory, attention or behavior problems were similar in both, the study group and the control group, but children exposed to cancer treatment during fetal life showed lower values of the visuospatial long-term memory than controls [93]. Multimodal MRI displayed less pronounced gyrification, especially in children exposed to intrauterine platinum derivates compared to controls, as well as lower fiber cross-section and lower fiber density in the posterior corpus callosum [94]. Nevertheless, brain organization and functional connectivity were not negatively affected by intrauterine exposure to chemotherapy or other oncological procedures [94].

Several studies investigated the outcome of children after intrauterine exposure to potentially cardiotoxic drugs, e.g., anthracyclines. No structural defects were observed during speckle-tracking echocardiographic examination in 90 children aged 5 years or older after intrauterine exposure to anthracyclines [95] and similar reassuring results were reported by Amant et al. in 69 children after intrauterine exposure to chemotherapy [77]. ECG measurements revealed no arrhythmia or conduction abnormalities [77]. In children exposed antenatally to anthracyclines, all cardiac dimensions, as well as systolic blood pressure, were within the normal range after a follow-up period of 22.3 months [77]. Ejection fraction, fractional shortening and interventricular septum thickness were still in the normal range but significantly decreased after anthracycline exposure compared to controls from a historical dataset [77]. Similar results were published previously [96]. Additionally, Vandenbroucke et al. found physical alterations in terms of higher diastolic blood pressure in children exposed to chemotherapy versus controls, but neither structural abnormalities nor rhythm or conduction abnormalities [93].

Cisplatin exposed children developed hearing loss in 3 of 8 children with available audiometric data [93] and similar effects were described in a previous case report [97].

## 4. Fertility Issues in Women with Cancer and Pregnancy

Being able to give birth is considered a basic human right and need and the possibility of being deprived of this option can lead to long-lasting traumatic events [98,99]. Thus, fertility concerns are frequently noticed in young women with cancer, as oncological treatment often affects the reproductive capability [100]. Schover et al. showed that 76% of 86 cancer patients of childbearing age without children at diagnosis wanted to have biological children after recovery from cancer [101,102]. Fertility issues and concerns do not necessarily stop with the birth of a child. Partridge et al. demonstrated that 63% of women with one prior live birth and up to 20% of women with two or more live births prior to cancer diagnosis quoted concerns regarding infertility as a possible side effect of cancer treatment [103].

There are no specific guidelines regarding fertility counseling and fertility preservation in women with cancer diagnosed during pregnancy. Partly due to lack of knowledge regarding fertility preservation methods and partly because of the ongoing pregnancy at time of diagnosis, some health care professionals may neglect the fact, that women who were diagnosed with cancer during pregnancy may yearn for more children after recovery [104]. Consequently, a large proportion of cancer patients of fertile age were not referred to a fertility specialists to discuss fertility issues before undergoing oncological treatment [104,105]. This had detrimental long-term effects on the psychological well-being of affected women [92]. According to current guidelines regarding fertility preservation of young oncological patients, all women who did not accomplish the family planning before treatment should be referred to a specialist for reproductive medicine for individual counseling [104,105,106].

Unfortunately, standard fertility preservation methods, such as cryo-preservation of fertilized or unfertilized oocytes, as well as cryo-preservation of ovarian tissue, are usually not feasible during pregnancy. However, other side effects of gonadotoxic treatment, such as symptoms of hormonal deficiency, sexual dysfunctions and mood disorders should be evaluated. After birth, an assessment of the residual reproductive capacity, as well as a re-evaluation of fertility preservation options should be offered [107], depending on the wishes of the women and on the current state of oncological treatment. In case of depletion of ovarian reserve due to cancer treatment and if oncologically safe, hormone replacement therapy should be initiated, as it reduces negative short-term and long-term effects of premature menopause, such as sleep disorders, mood changes and sexual problems [100].

## 5. Psychological Impact of Cancer in Pregnancy

Psychological changes are common in pregnant women. They often experience a mixture of feelings, such as happiness, enjoyment, delight or negative emotions such as anxiety, stress, fear and adaptive difficulties. The diagnosis of cancer bursts similarly to a bombshell in this sensible period. Cancer is first of all experienced as a life-threatening disease, regardless of the entity, stage or prognosis and the first thoughts of an expectant mother are towards their children and the implicated consequences, combined with feelings of guilt, grief, shame and loss of control [108]. Cancer comes unexpectedly and is never welcomed, especially considering pregnancy as a period of creation of a new life. The expectant mother is caught in an emotional chaos caused by fear for her own life and fear for the life and health of the unborn child, causing significantly distress [109]. Pregnant women seemed to be more susceptible to mood disorders than non-pregnant women [92]. Thus, a prompt assessment of mental health and referral to psychosocial services should be standard of care. Nevertheless, specific information about the psychological impact of cancer in pregnant women is scarce and facts about the psychological changes are often deduced from women suffering from cancer but who are not pregnant or from women who are pregnant but do not have cancer.

Non-pregnant women diagnosed with cancer showed high levels of psychosocial distress and post-traumatic stress disorders, depression and anxiety, during the early phases of diagnosis, as well as during cancer treatment and follow-up [110]. A meta-analysis published by Mitchell et al. showed a high prevalence of mood disorders in patients with cancer: depression occurred in 20.7% of cases, adjustment disorder in 31.6% and any type of mood disorder in 38.2% of patients [111]. Similar mood changes were observed in women diagnosed with cancer in pregnancy: 51.5% of pregnant women were clinically distressed, while the distress was mostly experienced as intrusive thoughts [92]. Positive coping strategies were associated with decreased level of concern [112]. Vandenbroucke et al. reported a high level of distress (in both parents) related to concerns regarding the child’s health, the cancer diagnosis and treatment, the outcome of the pregnancy and the delivery [113]. During the ongoing pregnancy, nulliparous women showed more concerns than multiparous, and in general, women were concerned about the effects of the chemotherapy on the progeny [112]. Pregnant women tended to overestimate the impact of antenatal chemotherapy on short-term and long-term effects on the offspring [112]. Additionally, a more advanced disease stage was correlated with increased concerns, but not with an increased wish for termination of pregnancy [112].

The postpartum period is often complicated by maternal mental disorders [114]. Depression was found in up to 24% of otherwise healthy postpartum mothers [114]. Risk factors for postpartum depression in women without a diagnosis of cancer in pregnancy were: delivery by cesarian section, the experience of other adverse birth or infant related factors, such as preterm birth or low-birth-weight infants, postpartum anemia, a history of mental disorders and perceived lack of social support [114]. Unfortunately, women diagnosed with cancer in pregnancy may meet several above-mentioned risk factors, and thus, are at high risk for developing postpartum depression or other mental health issues. Preterm birth is the most common obstetrical complication, as up to 50% of women diagnosed with cancer in pregnancy will experience a predominantly iatrogenic induced preterm delivery [8]. A recent meta-analysis confirmed the correlation between preterm delivery and depression with an OR 1.79 [115]. Additionally, anxiety symptoms were increased in parents with a child of very low birth weight [116]. Up to 41% of children of mothers with cancer in pregnancy were admitted to the NICU (neonatal intensive care unit) and NICU admission was associated with feelings of anxiety, distress, depression, powerlessness and hopelessness in otherwise healthy women [117]. Thus, counseling for distress should be offered to each woman diagnosed with cancer in pregnancy as well as to her partner.

## 6. Conclusions

Cancer during pregnancy is challenging for patients, their families and health care providers. Pregnancies complicated by a cancer diagnosis should be managed by a multidisciplinary team of specialists. Ideally, the diagnostic and therapeutic procedures in pregnant cancer patients should provide the same efficacy as in non-pregnant women and should not affect the development and long-term outcome of the unborn child. Surgery during pregnancy is a feasible option even in gynecological cancer and should be performed preferably within the second trimester. Most chemotherapeutical drugs can be safely applied after completion of organogenesis until 3 weeks prior to delivery. Radiotherapy should be scheduled for the period after delivery. Iatrogenic preterm delivery should be avoided, as it emerged to be the major risk factor for long-term adverse outcome of the offspring.

## Figures and Tables

**Table 1:** Stage-adapted treatment of breast cancer and gynecological cancer diagnosed during pregnancy.

Entity		Diagnostic Procedures	Tumor Stage	Possible Therapeutic Procedures During Pregnancy	Pregnancy and Delivery	Post Pregnancy
Breast Cancer		Breast US Axilla US Mammography Chest x-ray Liver US Bone MRI	Not locally advanced	1st trimester: consider TOP ^1^ prefer mastectomy over lumpectomy ^2^ Prefer SNLB of the axilla over axillary dissection ^2^ CT ^3^ RT ^4^ gw > 36: DTAD ^5^	OM ^5^	COT
			Locally advanced	1st trimester: consider TOP ^1^ prefer mastectomy over lumpectomy ^2^ Prefer SNLB of the axilla over axillary dissection ^2^ CT ^3^ RT ^4^ gw > 36: DTAD ^5^		
			Metastatic	1st trimester: consider TOP ^1^ CT ^3^ RT ^4^ gw > 36: DTAD ^5^		
Ovarian Cancer	Epithelial	gw < 22: LSC gw > 22: LAP biopsy or adenectomy	stage IA, IB	gw < 22, IAG1, no indication for CT, staging surgery without hysterectomy gw > 22, IAG1, no indication for CT, staging surgery after delivery gw > 22, indication for CT ^3^, staging surgery after delivery	OM ^5^	COT
			>stage IB	Consider TOP ^7^ NACT ^3^, staging surgery after delivery	OM ^6^	COT
	BOT	gw < 22: LSC gw > 22: LAP biopsy or adenectomy		conservative surgery ^2^	OM ^5^	COT
	Germ Cell	gw < 22: LSC gw > 22: LAP biopsy or adenectomy		if no indication for CT ^3^: follow up CT ^3^, if indicated	OM ^5^	COT
	Sex Cord	gw < 22: LSC gw > 22: LAP biopsy or adenectomy		gw < 22, no indication for CT, staging surgery without hysterectomy ^2^ gw > 22, no indication for CT, staging surgery after delivery CT ^3^, if indicated	OM ^5^	COT
Cervical Cancer		Colposcopy Biopsy MRI gw < 22: LSC, PLND ^2^	IA1, LVSI- IA1, LNM- IA2, LNM- IB1, LNM-	Conization ^2^ Simple trachelectomy ^2^ Radical trachelectomy ^2^ vs Consider NACT ^3^ vs gw > 22, DTAD can be considered	CS ^5^	COT
		Colposcopy Biopsy MRI gw < 22: LSC, PLND ^2^	IB2, LNM-	NACT ^3^ vs If gw > 22, DTAD can be considered	CS ^5^	COT
		Colposcopy Biopsy MRI	IB2, gw > 22, LVSI unkown, LNM unkown	NACT ^3^ vs DTAD	CS ^5^	COT
		Colposcopy Biopsy MRI	IB3	Consider TOP ^7^	CS ^6^	COT
				If TOP is not desired, discuss NACT ^3^	CS ^5^	
		Colposcopy Biopsy MRI	>IB3	TOP ^7^	CS ^6^	COT
				If TOP is not desired, discuss NACT ^3^	CS ^5^	
		Colposcopy Biopsy MRI gw < 22: LSC, PLND ^2^	All stages, LNM+	Consider TOP ^7^	CS ^6^	COT
				If TOP is not desired, discuss NACT ^3^	CS ^5^	
Vulvar cancer		Vulvoscopy Biopsy MRI	Early stages, no need for RT	Local excision ^2^ SNLB of the groin ^2^	CS ^5^	COT
			Advanced disease, need for RT	Consider delay of RT for 6 to 8 weeks	CS ^6^	COT
				RT cannot be delayed: TOP ^7^	CS ^6^	
Vaginal cancer		Vaginoscopy Biopsy MRI	Early stages, no need for RT	Local excision ^2^	CS ^5^	COT
			Advanced disease, need for RT	TOP ^7^	CS ^6^	COT

BOT = borderline tumor of the ovary; COT = competition of treatment, if necessary; CT = Chemotherapy; CS = cesarian section; DTAD = delay treatment after delivery; gw = gestational weeks; LSC = laparoscopy; LAP = laparotomy; LVSI = lymphovascular space invasion; LNM = lymph node metastases; MRI = magnetic resonance imaging; NACT = neoadjuvant chemotherapy, until disease progression is detected; OM = obstetrical management and delivery, according to respective guidelines for high-risk pregnancies; PLND = pelvic lymphadenectomy; RT = radiotherapy; SNLB = sentinel lymph node biopsy; TOP = termination of pregnancy; US = ultrasound; vs = versus; − = negative; + = positive; ^1^ = in cases requiring urgent systemic treatment, ^2^ = 2nd trimester recommended for surgical procedures; ^3^ = allowed in 2nd and 3rd trimester before 35 weeks of gestation; ^4^ = possible in selected cases during 1st and 2nd trimester; ^5^ = avoid iatrogenic preterm delivery; ^6^ = if fetus viable; ^7^ = consider feticide, if fetus not viable, with either vaginal or laparotomic evacuation of the uterus.

## References

[1] Peccatori F.A., Azim H.A., Orecchia R., Hoekstra H.J., Pavlidis N., Kesic V., Pentheroudakis G. (2013). Cancer, pregnancy and fertility: ESMO Clinical Practice Guidelines for diagnosis, treatment and follow-up. Ann. Oncol..

[2] Silverstein J., Post A.L., Chien A.J., Olin R., Tsai K.K., Ngo Z. (2020). Multidisciplinary management of cancer during pregnancy. JCO Oncol. Pract..

[3] Maggen C., Wolters V.E.R.A., Cardonick E., Fumagalli M., Halaska M.J., Lok C.A.R., De Haan J., Van Tornout K., Van Calsteren K., Amant F. (2020). Pregnancy and cancer: The INCIP project. Curr. Oncol. Rep..

[4] Bianchi D.W., Chudova D., Sehnert A.J., Bhatt S., Murray K., Prosen T.L., Garber J.E., Wilkins-Haug L., Vora N., Warsof S. (2015). Noninvasive prenatal testing and incidental detection of occult maternal malignancies. JAMA.

[5] Aktoz F., Yalcin A.C., Yüzdemir H.S., Akata D., Gültekin M. (2018). Treatment of massive liver metastasis of breast cancer during pregnancy: First report of a complete remission with trastuzumab and review of literature. J. Matern. Neonatal Med..

[6] Amant F., Halaska M.J., Fumagalli M., Steffensen K.D., Lok C., Van Calsteren K., Han S., Mir O., Fruscio R., Uzan C. (2014). Gynecologic cancers in pregnancy: Guidelines of a second international consensus meeting. Int. J. Gynecol. Cancer.

[7] Amant F., Berveiller P., Boere I., Cardonick E., Fruscio R., Fumagalli M., Halaska M., Hasenburg A., Johansson A., Lambertini M. (2019). Gynecologic cancers in pregnancy: Guidelines based on a third international consensus meeting. Ann. Oncol..

[8] de Haan J., Verheecke M., Van Calsteren K., Van Calster B., Shmakov R.G., Gziri M.M. (2018). Oncological management and obstetric and neonatal outcomes for women diagnosed with cancer during pregnancy: A 20-year international cohort study of 1170 patients. Lancet Oncol..

[9] Vandecaveye V., Amant F., Lecouvet F., Van Calsteren K., Dresen R.C. (2021). Imaging modalities in pregnant cancer patients. Int. J. Gynecol. Cancer.

[10] Wieseler K.M., Bhargava P., Kanal K.M., Vaidya S., Stewart B.K., Dighe M.K. (2010). Imaging in pregnant patients: Examination appropriateness. Radiographics.

[11] Austin L.M., Frush D.P. (2011). Compendium of national guidelines for imaging the pregnant patient. Am. J. Roentgenol..

[12] Coakley F.V., Cody D.D., Mahesh M. (2010). The pregnant patient: Alternatives to CT and dosesaving modifications to CT technique. Image Wisely.

[13] Mazzola R., Corradini S., Eidemüeller M., Figlia V., Fiorentino A., Giaj-Levra N., Nicosia L., Ricchetti F., Rigo M., Musola M. (2019). Modern radiotherapy in cancer treatment during pregnancy. Crit. Rev. Oncol..

[14] Potts J., Lowe S.A. (2020). Imaging in pregnancy stewardship. Obstet. Med..

[15] Han S.N., Verheecke M., Amant T.V., Gziri M.M., Van Calsteren K., Amant F. (2014). Management of gynecological cancers during pregnancy. Curr. Oncol. Rep..

[16] Shachar S.S., Gallagher K., McGuire K., Zagar T.M., Faso A., Muss H.B., Sweeting R., Anders C.K. (2018). Multidisciplinary management of breast cancer during pregnancy. Oncologist.

[17] Loibl S., Schmidt A., Gentilini O.D., Kaufman B., Kuhl C., Denkert C., Von Minckwitz G., Parokonnaya A., Stensheim H., Thomssen C. (2015). Breast cancer (diagnosed) during pregnancy: Adapting recent advances in breast cancer care for pregnant patients. JAMA Oncol..

[18] Paris I., Di Giorgio D., Carbognin L., Corrado G., Garganese G., Franceschini G., Sanchez A.M., De Vincenzo R.P., Accetta C., Terribile D.A. (2021). Pregnancy-associated breast cancer: A multidisciplinary approach. Clin. Breast Cancer.

[19] Behtash N., Zarchi M.K., Gilani M.M., Ghaemmaghami F., Mousavi A., Ghotbizadeh F. (2008). Ovarian carcinoma associated with pregnancy: A clinicopathologic analysis of 23 cases and review of the literature. BMC Pregnancy Childbirth.

[20] Wang L., Huang S., Sheng X., Ren C., Wang Q., Yang L., Zhao S., Xu T., Ma X., Guo R. (2020). Malignant ovarian tumors during pregnancy: A multicenter retrospective analysis. Cancer Manag. Res..

[21] Zanotti K.M., Belinson J.L., Kennedy A.W. (2000). Treatment of gynecologic cancers in pregnancy. Semin. Oncol..

[22] Zilliox M., Lecointre L., Azais H., Ballester M., Bendifallah S., Bolze P., Bourdel N., Bricou A., Canlorbe G., Carcopino X. (2021). Management of borderline ovarian tumours during pregnancy: Results of a French multi-centre study. Eur. J. Obstet. Gynecol. Reprod. Biol..

[23] Nasioudis D., Mulugeta-Gordon L., McMinn E., Byrne M., Ko E.M., Cory L., Latif N.A. (2021). Oncologic outcomes of uterine preservation for pre-menopausal patients with stage II epithelial ovarian carcinoma. Int. J. Gynecol. Cancer.

[24] Schuurman T., Zilver S., Samuels S., Schats W., Amant F., van Trommel N., Lok C. (2021). Fertility-Sparing Surgery in Gynecologic Cancer: A Systematic Review. Cancers.

[25] Korenaga T.-R.K., Tewari K.S. (2020). Gynecologic cancer in pregnancy. Gynecol. Oncol..

[26] Dłuski D.F., Mierzyński R., Poniedziałek-Czajkowska E., Leszczyńska-Gorzelak B. (2020). Ovarian cancer and pregnancy—A current problem in perinatal medicine: A Comprehensive Review. Cancers.

[27] Zong X., Yang J.X., Zhang Y., Cao D.Y., Shen K. (2020). Clinicopathological characteristics and treatment outcomes of pregnancy complicated by malignant ovarian germ cell tumors. Cancer Manag. Res..

[28] Michalczyk K., Cymbaluk-Płoska A. (2021). Approaches to the diagnosis and management of ovarian cancer in pregnancy. Cancer Manag. Res..

[29] Beharee N., Shi Z., Wu D., Wang J. (2019). Diagnosis and treatment of cervical cancer in pregnant women. Cancer Med..

[30] Martinelli F., Ditto A., Filippi F., Vinti D., Bogani G., Maggiore U.L.R., Evangelista M., Signorelli M., Chiappa V., Lopez S. (2021). Conization and lymph node evaluation as a fertility-sparing treatment for early stage cervical cancer. Int. J. Gynecol. Cancer.

[31] Fanfani F., Anchora L.P., Di Martino G., Bizzarri N., Di Meo M.L., Carbone V., Paderno M., Fedele C., Paniga C., Fagotti A. (2021). Oncologic and obstetric outcomes after simple conization for fertility-sparing surgery in FIGO 2018 stage IB1 cervical cancer. Int. J. Gynecol. Cancer.

[32] Song Y., Liu Y., Lin M., Sheng B., Zhu X. (2018). Efficacy of neoadjuvant platinum-based chemotherapy during the second and third trimester of pregnancy in women with cervical cancer: An updated systematic review and meta-analysis. Drug Des. Dev. Ther..

[33] Arakawa A., Ichikawa H., Kubo T., Motoi N., Kumamoto T., Nakajima M., Yonemori K., Noguchi E., Sunami K., Shiraishi K. (2021). Vaginal transmission of cancer from mothers with cervical cancer to infants. N. Engl. J. Med..

[34] Koren G., Pariente G. (2018). Pregnancy-associated changes in pharmacokinetics and their clinical implications. Pharm. Res..

[35] Amant F., von Minckwitz G., Han S.N., Bontenbal M., Ring A.E., Giermek J., Loibl S. (2013). Prognosis of women with primary breast cancer diagnosed during pregnancy: Results from an international collaborative study. J. Clin. Oncol..

[36] Cardonick E., Eicheldinger E., Gaughan J.P. (2019). Chemotherapy is avoided during the first trimester of pregnancy, when is the safest time to start treatment during the second or third trimester?. ProClinS Gynecol. Obstet..

[37] Gouveia H.J., Manhães-De-Castro R., Costa-De-Santana B.J., Mendonça C.R., Albuquerque G., Visco D.B., Lacerda D.C., Toscano A.E. (2020). Maternal exposure to busulfan reduces the cell number in the somatosensory cortex associated with delayed somatic and reflex maturation in neonatal rats. J. Chem. Neuroanat..

[38] Anderka M., Mitchell A.A., Louik C., Werler M.M., Hernández-Diaz S., Rasmussen S.A. (2012). Medications used to treat nausea and vomiting of pregnancy and the risk of selected birth defects. Birth Defects Res. Part A Clin. Mol. Teratol..

[39] Pasternak B., Svanström H., Mølgaard-Nielsen D., Melbye M., Hviid A. (2013). Metoclopramide in pregnancy and risk of major congenital malformations and fetal death. JAMA.

[40] Oliveira L.G., Capp S.M., You W.B., Riffenburgh R.H., Carstairs S.D. (2014). Ondansetron compared with doxylamine and pyridoxine for treatment of nausea in pregnancy: A randomized controlled trial. Obstet. Gynecol..

[41] Rodríguez-Pinilla E., Martínez-Frías M.L. (1998). Corticosteroids during pregnancy and oral clefts: A case-control study. Teratology.

[42] Boxer L.A., Bolyard A.A., Kelley M.L., Marrero T.M., Phan L., Bond J.M., Newburger P.E., Dale D.C. (2015). Use of granulocyte colony-stimulating factor during pregnancy in women with chronic neutropenia. Obstet. Gynecol..

[43] Federal Register Content and Format of Labeling for Human Prescription Drug and Biological Products. https://www.federalregister.gov/documents/2014/12/04/2014–28241/content-and-format-of-labeling-for-human-prescription-drug-and-biological-products-requirements-for..

[44] Describing Medicines in Pregnancy Database. https://www.tga.gov.au/prescribing-medicines-pregnancy-database..

[45] Embryotox—Arzneimittelsicherheit in Schwangerschaft und Stillzeit. https://www.embryotox.de/.

[46] Sarno M.A., Mancari R., Azim H.A., Colombo N., Peccatori F.A. (2013). Are monoclonal antibodies a safe treatment for cancer during pregnancy?. Immunotheraphy.

[47] Lambertini M., Peccatori F.A., Azim H.A. (2015). Targeted agents for cancer treatment during pregnancy. Cancer Treat. Rev..

[48] Zagouri F., Sergentanis T.N., Chrysikos D., Papadimitriou C.A., Dimopoulos M.A., Bartsch R. (2013). Trastuzumab administration during pregnancy: A systematic review and meta-analysis. Breast Cancer Res. Treat..

[49] Lambertini M., Martel S., Msc C.C., Msc S.G., Msc F.S.H., Schuehly U., Korde L., Azim H.A., Di Cosimo S., Tenglin R.C. (2019). Pregnancies during and after trastuzumab and/or lapatinib in patients with human epidermal growth factor receptor 2-positive early breast cancer: Analysis from the NeoALTTO (BIG 1-06) and ALTTO (BIG 2-06) trials. Cancer.

[50] Chakravarty E.F., Murray E.R., Kelman A., Farmer P. (2011). Pregnancy outcomes after maternal exposure to rituximab. Blood.

[51] Lishner M., Avivi I., Apperley J.F., Dierickx D., Evens A.M., Fumagalli M., Nulman I., Oduncu F.S., Peccatori F.A., Robinson S. (2016). Hematologic malignancies in pregnancy: Management guidelines from an international consensus meeting. J. Clin. Oncol..

[52] Yockey L.J., Iwasaki A., Haven N., Chase C. (2018). HHS public access. Immunity.

[53] Ringley J.T., Moore D.C., Patel J., Rose M.S. (2018). Poly (ADP-ribose) polymerase inhibitors in the management of ovarian cancer: A drug class review. Pharm. Ther..

[54] Johnson D.B., Sullivan R.J., Menzies A.M. (2018). Immune checkpoint inhibitors in challenging populations. Cancer.

[55] Wolters V., Heimovaara J., Maggen C., Cardonick E., Boere I., Lenaerts L., Amant F. (2021). Management of pregnancy in women with cancer. Int. J. Gynecol. Cancer.

[56] Burotto M., Gormaz J.G., Samtani S., Valls N., Silva R., Rojas C., Portiño S., de la Jara C. (2018). Viable pregnancy in a patient with metastatic melanoma treated with double checkpoint immunotherapy. Semin. Oncol..

[57] Braems G., Denys H., De Wever O., Cocquyt V., Van den Broecke R. (2011). Use of tamoxifen before and during pregnancy. Oncologist.

[58] Tiboni G.M., Ponzano D.A. (2016). Fetal safety profile of aromatase inhibitors: Animal data. Reprod. Toxicol..

[59] Mitwally M.F., Hozayen W.G., Hassanin K.M., Abdalla K.A., Abdalla N.K. (2020). Aromatase inhibitor letrozole: A novel treatment for ectopic pregnancy. Fertil. Steril..

[60] Antolak J.A., Strom E.A., Antolak J.A., Strom E.A. (2004). Fetal dose estimates for electron-beam treatment to the chest wall of a pregnant patient Fetal dose estimates for electron-beam treatment to the chest wall of a pregnant patient. Med. Phys..

[61] Candela-Juan C., Gimeno-Olmos J., Pujades M.C., Rivard M.J., Carmona V., Lliso F., Perez-Calatayud J. (2015). Fetal dose measurements and shielding efficiency assessment in a custom setup of 192Ir brachytherapy for a pregnant woman with breast cancer. Phys. Med..

[62] Kourinou K.M., Mazonakis M., Lyraraki E., Damilakis J. (2015). Photon-beam radiotherapy in pregnant patients: Can the fetal dose be limited to 10 cGy or less?. Phys. Med..

[63] Ngu S.L., Duval P., Collins C. (1992). Foetal radiation dose in radiotherapy for breast cancer. Australas. Radiol..

[64] Stensheim H., Møller B., Van Dijk T., Fosså S.D. (2009). Cause-specific survival for women diagnosed with cancer during pregnancy or lactation: A registry-based cohort study. J. Clin. Oncol..

[65] Boudy A.S., Ferrier C., Selleret L., Zilberman S., Arfi A., Sussfeld J., Gligorov J., Richard S., Bendifallah S., Chabbert-Buffet N. (2020). Prognosis of HER2-positive pregnancy-associated breast cancer: Analysis from the French CALG (Cancer Associé à La Grossesse). Breast.

[66] Azim H.A., Santoro L., Russell-Edu W., Pentheroudakis G., Pavlidis N., Peccatori F.A. (2012). Prognosis of pregnancy-associated breast cancer: A meta-analysis of 30 studies. Cancer Treat. Rev..

[67] Shao C., Yu Z., Xiao J., Liu L., Hong F., Zhang Y., Jia H. (2020). Prognosis of pregnancy-associated breast cancer: A meta-analysis. BMC Cancer.

[68] Maggen C., Dierickx D., Lugtenburg P., Laenen A., Cardonick E., Smakov R.G., Bellido M., Cabrera-Garcia A., Gziri M.M., Halaska M.J. (2019). Obstetric and maternal outcomes in patients diagnosed with Hodgkin lymphoma during pregnancy: A multicentre, retrospective, cohort study. Lancet Haematol..

[69] Perrone A.M., Livi A., Fini M., Bondioli E., Concetti S., Morganti A.G., Contedini F., De Iaco P. (2016). A surgical multi-layer technique for pelvic reconstruction after total exenteration using a combination of pedicled omental flap, human acellular dermal matrix and autologous adipose derived cells. Gynecol. Oncol. Rep..

[70] Abellar R.G., Pepperell J.R., Greco D., Gundogan F., Schwartz J., Tantravahi U., De Paepe M.E., Kostadinov S. (2009). Effects of chemotherapy during pregnancy on the placenta. Pediatric Dev. Pathol..

[71] Verheecke M., Calabuig A.C., Ferreiro J.F., Brys V., Van Bree R., Verbist G., Everaert T., Leemans L., Gziri M., Boere I. (2018). Genetic and microscopic assessment of the human chemotherapy-exposed placenta reveals possible pathways contributive to fetal growth restriction. Placenta.

[72] Eliesen G.A.M., van Hove H., Meijer M.H., Broek P.H.H.V.D., Pertijs J., Roeleveld N., van Drongelen J., Russel F.G.M., Greupink R. (2021). Toxicity of anticancer drugs in human placental tissue explants and trophoblast cell lines. Arch. Toxicol..

[73] Bar-Joseph H., Peccatori F.A., Goshen-Lago T., Cribiù F.M., Scarfone G., Miller I., Nemerovsky L., Levi M., Shalgi R., Ben-Aharon I. (2020). Cancer during pregnancy: The role of vascular toxicity in chemotherapy-induced placental toxicity. Cancers.

[74] Pacifici G.M., Nottoli R. (1995). Placental transfer of drugs administered to the mother. Clin. Pharmacokinet..

[75] Van Calsteren K., Verbesselt R., Van Bree R., Heyns L., De Bruijn E., De Hoon J., Amant F. (2010). Substantial variation in transplacental transfer of chemotherapeutic agents in a mouse model. Reprod. Sci..

[76] Cardonick E., Iacobucci A. (2004). Use of chemotherapy during human pregnancy. Lancet Oncol..

[77] Amant F., Van Calsteren K., Halaska M.J., Gziri M.M., Hui W., Lagae L., Willemsen M., Kapusta L., Van Calster B., Wouters H. (2012). Long-term cognitive and cardiac outcomes after prenatal exposure to chemotherapy in children aged 18 months or older: An observational study. Lancet Oncol..

[78] Amant F., Vandenbroucke T., Verheecke M., Fumagalli M., Halaska M.J., Boere I., Han S., Gziri M.M., Peccatori F., Rob L. (2015). Pediatric outcome after maternal cancer diagnosed during pregnancy. N. Engl. J. Med..

[79] Amant F., Loibl S., Neven P., Van Calsteren K. (2012). Breast cancer in pregnancy. Lancet.

[80] Stopenski S., Aslam A., Zhang X., Cardonick E. (2017). After chemotherapy treatment for maternal cancer during pregnancy, is breastfeeding possible?. Breastfeed. Med..

[81] Cooke M., Sheehan A., Schmied V. (2003). A description of the relationship between breastfeeding experiences, breastfeeding satisfaction, and weaning in the first 3 months after birth. J. Hum. Lact..

[82] Martinez M.E., Wertheim B.C., Natarajan L., Schwab R., Bondy M., Daneri-Navarro A., Meza-Montenegro M.M., Gutierrez-Millan L.E., Brewster A., Komenaka I.K. (2013). Reproductive factors, heterogeneity, and breast tumor subtypes in women of mexican descent. Cancer Epidemiol. Biomark. Prev..

[83] Kalarikkal S., Pfleghaar J. (2020). Breastfeeding. StatPearls [Internet].

[84] Durodola J.I. (1979). Administration of cyclophosphamide during late pregnancy and early lactation: A case report. J. Natl. Med Assoc..

[85] Pistilli B.B., Bellettini G.G., Giovannetti E., Codacci-Pisanelli G.G., Azim H.H.A., Benedetti G.G., Sarno M.A.M., Peccatori F.A. (2013). Chemotherapy, targeted agents, antiemetics and growth-factors in human milk: How should we counsel cancer patients about breastfeeding?. Cancer Treat. Rev..

[86] Russell M.A., Carpenter M.W., Akhtar M.S., Lagattuta T.F., Egorin M.J. (2007). Imatinib mesylate and metabolite concentrations in maternal blood, umbilical cord blood, placenta and breast milk. J. Perinatol..

[87] Terao R., Nii M., Asai H., Nohara F., Okamoto T., Nagaya K., Azuma H. (2020). Breastfeeding in a patient with chronic myeloid leukemia during tyrosine kinase inhibitor therapy. J. Oncol. Pharm. Pract..

[88] Gambacorti-Passerini C.B., Tornaghi L., Marangon E., Franceschino A., Pogliani E.M., D’Incalci M., Zucchetti M. (2007). Imatinib concentrations in human milk. Blood.

[89] Cardonick E., Dougherty R., Grana G., Gilmandyar D., Ghaffar S., Usmani A. (2010). Breast cancer during pregnancy maternal and fetal outcomes. Cancer J..

[90] Cardonick E., Usmani A., Ghaffar S. (2010). Perinatal outcomes of a pregnancy complicated by cancer, including neonatal follow-up after in utero exposure to chemotherapy: Results of an international registry. Am. J. Clin. Oncol. Cancer Clin. Trials.

[91] Azim H.A., Santoro L., Pavlidis N., Peccatori F.A. (2010). Safety of pregnancy in breast cancer survivors: A meta-analysis. Eur. J. Cancer.

[92] Henry M., Huang L.N., Sproule B.J., Cardonick E. (2011). The psychological impact of a cancer diagnosed during pregnancy: Determinants of long-term distress. Psycho Oncol..

[93] Vandenbroucke T., Verheecke M., van Gerwen M., Van Calsteren K., Halaska M.J., Fumagalli M., Fruscio R., Gandhi A., Veening M., Lagae L. (2020). Child development at 6 years after maternal cancer diagnosis and treatment during pregnancy. Eur. J. Cancer.

[94] Blommaert J., Radwan A., Sleurs C., Maggen C., van Gerwen M., Wolters V., Christiaens D., Peeters R., Dupont P., Sunaert S. (2020). The impact of cancer and chemotherapy during pregnancy on child neurodevelopment: A multimodal neuroimaging analysis. EClinicalMedicine.

[95] Avilès A., Nambo M.-J., Huerta-Guzmàn J., Neri N., Cleto S. (2016). Speckle-tracking echocardiography to detect cardiac toxicity in children who received anthracyclines during pregnancy. Clin. Lymphoma Myeloma Leuk..

[96] Gziri M.M., Hui W., Amant F., Van Calsteren K., Ottevanger N., Kapusta L., Mertens L. (2013). Myocardial function in children after fetal chemotherapy exposure: A tissue Doppler and myocardial deformation imaging study. Eur. J. Pediatrics.

[97] Geijteman E.C., Wensveen C.W., Duvekot J.J., Van Zuylen L. (2014). A child with severe hearing loss associated with maternal cisplatin treatment during pregnancy. Obstet. Gynecol..

[98] Ussher J.M., Perz J. (2018). Threat of biographical disruption: The gendered construction and experience of infertility following cancer for women and men. BMC Cancer.

[99] Ussher J.M., Cummings J.R., Dryden A., Perz J. (2016). Talking about fertility in the context of cancer: Health care professional perspectives. Eur. J. Cancer Care.

[100] Dittrich R., Kliesch S., Schüring A., Balcerek M., Baston-Büst D.M., Beck R., Beckmann M.W., Behringer K., Borgmann-Staudt A., Cremer W. (2018). Fertility preservation for patients with malignant disease. Guideline of the DGGG, DGU and DGRM (S2k-Level, AWMF Registry No. 015/082, November 2017)—Recommendations and Statements for Girls and Women. Geburtshilfe Frauenheilkd..

[101] Schover L.R. (2009). Patient attitudes toward fertility preservation. Pediatr. Blood Cancer.

[102] Schover L., Rybicki L.A., Martin B.A., Bringelsen K.A. (1999). Having children after cancer: A pilot survey of survivors’ attitudes and experiences. Cancer.

[103] Partridge A.H., Gelber S., Peppercorn J., Sampson E., Knudsen K., Laufer M., Rosenberg R., Przypyszny M., Rein A., Winer E.P. (2004). Web-based survey of fertility issues in young women with breast cancer. J. Clin. Oncol..

[104] Schwab R., Kiemen A., Weis J., Hasenburg A. (2020). Sexual Health, Fertility, And Relationships in Cancer Care: Provision of Onco-Fertility Support.

[105] Donnez J., Dolmans M.-M. (2017). Fertility preservation in women. New Engl. J. Med..

[106] Lambertini M., Peccatori F., Demeestere I., Amant F., Wyns C., Stukenborg J.-B., Paluch-Shimon S., Halaska M., Uzan C., Meissner J. (2020). Fertility preservation and post-treatment pregnancies in post-pubertal cancer patients: ESMO Clinical Practice Guidelines. Ann. Oncol..

[107] Anderson R.A., Amant F., Braat D., D’Angelo A., Lopes S.M.C.D.S., Demeestere I., Dwek S., Frith L., Lambertini M., The ESHRE Guideline Group on Female Fertility Preservation (2020). ESHRE guideline: Female fertility preservation. Hum. Reprod. Open.

[108] Kuswanto C.N., Stafford L., Sharp J., Schofield P. (2018). Psychological distress, role, and identity changes in mothers following a diagnosis of cancer: A systematic review. Psycho-Oncology.

[109] Faccio F., Mascheroni E., Ionio C., Pravettoni G., Peccatori F.A., Pisoni C., Cassani C., Zambelli S., Zilioli A., Nastasi G. (2020). Motherhood during or after breast cancer diagnosis: A qualitative study. Eur. J. Cancer Care.

[110] Rubin G., Berendsen A., Crawford S.M., Dommett R., Earle C., Emery J., Fahey T., Grassi L., Grunfeld E., Gupta S. (2015). The expanding role of primary care in cancer control. Lancet Oncol..

[111] Mitchell A.J., Chan M., Bhatti H., Halton M., Grassi L., Johansen C., Meader N. (2011). Prevalence of depression, anxiety, and adjustment disorder in oncological, haematological, and palliative care settings: A meta-analysis of 94 interview-based studies. Lancet Oncol..

[112] Leung V., Bryant C., Stafford L. (2020). Psychological aspects of gestational cancer: A systematic review. Psycho-Oncology.

[113] Vandenbroucke T., Han S.N., Van Calsteren K., Wilderjans T.F., Bergh B.R.H.V.D., Claes L., Amant F. (2016). Psychological distress and cognitive coping in pregnant women diagnosed with cancer and their partners. Psycho-Oncology.

[114] Zhao X.H., Zhang Z.H. (2020). Risk factors for postpartum depression: An evidence-based systematic review of systematic reviews and meta-analyses. Asian J. Psychiatr..

[115] de Paula Eduardo J.A.F., de Rezende M.G., Menezes P.R., Del-Ben C.M. (2019). Preterm birth as a risk factor for postpartum depression: A systematic review and meta-analysis. J. Affect. Disord..

[116] Helle N., Barkmann C., Ehrhardt S., Von Der Wense A., Nestoriuc Y., Bindt C. (2016). Postpartum anxiety and adjustment disorders in parents of infants with very low birth weight: Cross-sectional results from a controlled multicentre cohort study. J. Affect. Disord..

[117] Roque A.T.F., Lasiuk G.C., Radünz V., Hegadoren K. (2017). Scoping review of the mental health of parents of infants in the NICU. J. Obstet. Gynecol. Neonatal Nurs..

